# PCDHGB7 Increases Chemosensitivity to Carboplatin by Inhibiting HSPA9 via Inducing Apoptosis in Breast Cancer

**DOI:** 10.1155/2019/6131548

**Published:** 2019-07-08

**Authors:** Siqi Hou, Ming Shan, Chunyang Gao, Xinxin Feng, Yongheng Yang, Ruo Zhang, Yan He, Guoqiang Zhang, Lei Zhang

**Affiliations:** ^1^Department of Pathology, Basic Medical Science College, Harbin Medical University, Harbin, China; ^2^Department of BC Surgery, Harbin Medical University Cancer Hospital, Harbin, China; ^3^Shuwen Biotech Co. Ltd., Deqing, China

## Abstract

Breast cancer is one of the most serious cancers worldwide, and chemotherapy resistance frequently drives cancer progression. Triple-negative breast cancer (TNBC) has a high recurrence rate and poor prognosis given its resistance to chemotherapy. In our previous study, we found a remarkable abnormal methylation modification of the PCDHGB7 gene in breast cancer. However, the roles of PCDHGB7 in the progression and treatment of breast cancer are unclear. In this study, we examined the effects of PCDHGB7 on the sensitivity of TNBC cells to carboplatin and investigated the underlying mechanism. By knocking down and overexpressing PCDHGB7 in HS578T and BT549 cells, we confirmed that PCDHGB7 increases TNBC cell chemosensitivity to carboplatin. Mechanistically, we found that PCDHGB7 negatively regulates the expression of HSPA9, uplifting its inhibition on P53 translocation and caspase-3 activation. Thus, we demonstrated that PCDHGB7 increases chemosensitivity of TNBC cells to carboplatin by inhibiting HSPA9 via inducing apoptosis. PCDHGB7 and HSPA9 represent potential therapeutic targets for chemosensitivity in breast cancer.

## 1. Introduction

Breast cancer is one of the leading causes of death among women worldwide and is the second leading cause of cancer mortality in women according to the American Cancer Society [1]. Chemoresistance leads to unsatisfactory treatment of breast cancer and disease progression. Triple-negative breast cancer (TNBC) is a breast cancer subtype that lacks the expression of three receptors (ER, PR, and HER2). TNBC exhibits a high recurrence rate and poor prognosis given its resistance to chemotherapy [2]. Correlation studies on chemotherapy sensitivity and resistance have thus far focused on DNA repair, ATP-binding cassette (ABC) transporter proteins, mitochondrial alteration, autophagy, epithelial-mesenchymal transition, tumor suppressor gene [3, 4], and glucose metabolism [5]. However, many questions regarding chemoresistance in TNBC remain unresolved.

PCDHGB7 is a member of the protocadherins, which have been demonstrated to inhibit tumorigenesis and cancer progression by inducing cell cycle arrest and apoptosis. Previous studies confirmed that PCDHGB7 plays roles in the process of self-recognition and mutual recognition between synapses, the movement of synapses, and the establishment of the nervous system network [6–9]. In humans, PCDHGB7 is predominantly expressed in the brain, spleen, heart, endometrium, esophagus, gall bladder, urinary bladder, and prostate. Various protocadherins play an inhibitory role in tumor genesis via various pathways, including promotion of cell cycle arrest and induction of apoptosis [10]. In one study, PCDHGB7 was reported to be significantly methylated in non-Hodgkin's lymphoma [11], whereas PCDHGB7 expression in other tumors and its role and mechanism in tumorigenesis and progression have not been reported. Our group analyzed and obtained a large number of promoter hypermethylation genes in breast cancer from the TCGA database in an early study of screening diagnostic markers for the methylation of breast cancer [10, 12]. Among these genes, abnormal methylation of the PCDHGB7 gene was seen in approximately 80% of breast cancer. Our group also detected PCDHGB7 mRNA expression in 40 paired breast cancer tissues and matched normal breast tissues and found PCDHGB7 expression was reduced in breast cancer tissue. Similar results were also obtained in breast cancer cell lines by our group, suggesting that PCDHGB7 gene expression is significantly reduced in breast cancer due to hypermethylation of the promoter region. We demonstrated that PCDHGB7 also acts as a tumor suppressor in breast cancer by enhancing the proliferation and invasion of breast carcinoma cell lines. In this study, we seek to assess the role of PCDHGB7 in the chemoresistance of TNBC and its corresponding mechanism.

Our preliminary investigation suggested that HSPA9 (heat shock protein family A (Hsp70) member 9) expression is highly correlated with PCDHGB7 gene expression and that PCDHGB7 knockdown induces significant upregulation of HSPA9 in a BC cell line. As a member of the HSP70 family, HSPA9 is also known as mortalin [13]. It plays multiple roles in mitochondrial import, intracellular transport, energy generation, and stress responses [14–17]. Recently, many studies have confirmed that HSPA9 promotes the occurrence and progression of cancer by promoting tumor cell proliferation, inhibiting tumor cell apoptosis, and promoting angiogenesis [18–20].

In this study, by constructing PCDHGB7 knockdown and overexpression models and HSPA9 knockdown breast carcinoma cell models, we confirmed the positive correlation between the PCDHGB7 level and the cell's chemosensitivity to carboplatin. Our results demonstrated that PCDHGB7 inhibits HSPA9 and increases chemosensitivity to carboplatin via inducing apoptosis in breast cancer. PCDHGB7 and HSPA9 potentially represent novel targets of chemotherapy in breast cancer.

## 2. Materials and Methods

### 2.1. Cell Lines and Cell Culture

TNBC cell lines (Hs578T and BT549) were obtained from the Affiliated Tumor Hospital of Harbin Medical University. Both cell lines were grown in Dulbecco's modified Eagles medium (Sigma-Aldrich, St. Louis, MO, USA) supplemented with 10% FBS (Gibco, USA) at 37°C with 5% CO_2_.

### 2.2. Total RNA Extraction and Quantitative Real-Time PCR

Trizol reagent (Invitrogen, USA) was used to obtain total RNA. The TransScript® One-Step gDNA Removal and cDNA Synthesis SuperMix Kit (Trans®, China) were employed to obtain cDNA by reverse transcription (RT). qRT-PCR was performed subsequently using an ABI 7900 RT-PCR system and TransScript® Tip Green qPCR SuperMix Kit (Trans®, China) according to the manufacturer's protocol. The following primers were synthesized by GenePharma (Shanghai, China): PCDHGB7, 5′-ACAGGGTTAGCCTTCGGGAA-3′ (forward) and 5′-ATCCTCGGTCTTTTGCTTCTATG-3′ (reverse); GAPDH, 5′-TGTGGGCATCAATGGATTTGG-3 (forward) and 5′-ACACCATGTATTCCGGGTCAAT-3′ (reverse); and HSPA9, 5′-CTTGTTTCAAGGCGGGATTATGC-3 (forward) and 5′-ACACCATGTATTCCGGGTCAAGCAGGAGTTGGTAGTACCCAAAT-3′ (reverse). GAPDH mRNA was used as an internal reference. PCR amplification was carried out using the following conditions: (1) initial denaturation: 94°C for 30 s, (2) denaturation: 94°C for 5 s, (3) annealing: 55°C for 15 s, and (4) extension: 72°C for 10 s. The PCR included 45 cycles of amplification and was followed by a melting curve analysis. Data were assessed using the by 2^-*ΔΔ*Ct^ method. All experiments were performed in triplicate.

### 2.3. Transfection

pLVX lentiviral vectors (Addgene, USA) were employed to overexpress PCDHGB7 in Hs578T and BT549 cell lines, and eLCv2 lentiviral vectors (Addgene, USA) were employed to knockdown PCDHGB7 in Hs578T and BT549 cell lines. To construct the plasmid overexpressing PCDHGB7, the full-length sequence of PCDHGB7 was amplified by RT-PCR and was inserted into the pLVX lentiviral vector. The sequences were designed to knockdown PCDHGB7 using the eLCv2 plasmid. The sequences were as follows: PCDHGB7sg-1, CTAAGGCGGTCAGTACCAAG; PCDHGB7sg-2, CTACAAGCTAGTAACAGATG. The sequences were designed to knockdown HSPA9 in PCDHGB7 cell lines using the eLCv2 plasmid. The sequences were as follows: HSPA9sg-1, ATCACTGAAGCTGTTCGAAAT; HSPA9sg-2, ACCCAAGCATTTGAATATGAA. To pack PCDHGB7 overexpression and knockdown lentiviral plasmids, the plasmids were transfected into HEK-293TN cells together with the packaging plasmids (pSPAX2 and pMD.2G) for 48 h. Then, the supernatant was harvested and used to infect Hs578T and BT549 cell lines. The same procedure was used for HSPA9 knockdown cell lentiviral plasmids.

### 2.4. Western Blot

Transfected Hs578T and BT549 cell lines were lysed with RIPA buffer, and total protein was isolated and quantitated using a BCA protein assay kit (Beyotime, China). The proteins were resolved via SDS-PAGE and then transferred onto PVDF membranes (Bio-Rad, USA). The membranes were incubated with mouse monoclonal anti-human HSPA9 (ab2799, Abcam, USA, 1 : 1000), rabbit polyclonal anti-active caspase-3 (ab2302, Abcam, USA), and rabbit monoclonal anti-human GAPDH antibodies (Abcam, USA). Then, the membranes were washed and incubated with horseradish peroxidase-conjugated secondary goat anti-rabbit for 1 hour. Protein expression was assessed using the ECL kit (Beyotime, China). The same protocol was performed to obtain protein from mouse tissue.

### 2.5. Chemosensitivity Assay

The Cell Counting Kit- (CCK-) 8 (K1018, APEXBIO, Shanghai, China) was used to assess the effect of the cell survival after treatment with carboplatin. Briefly, 5 × 10^3^ cells were seeded per well in 0.1 mL Dulbecco's modified Eagles medium (Sigma-Aldrich) supplemented with 10% FBS (Gibco, USA). Cells were treated with various concentrations of carboplatin 24 hours after transfection in cases of overexpression and 48 hours after transfection in cases of knockdown. After treatment, cells were incubated with CCK-8 dye for 1 hour. Absorbance was measured at 490 nm using a spectrophotometer (Molecular Devices, America).

### 2.6. Statistical Analysis

Statistical comparisons between the treatment group and control were performed using one-way analysis of variance (ANOVA) and Student's *t*-test. Data are expressed as the mean ± SEM. Differences were considered significant at *P* < 0.05. All statistical analyses were performed using Prism 6.0 (La Jolla, CA, USA).

## 3. Results

### 3.1. PCDHGB7 Significantly Enhances the Chemosensitivity of Hs578T and BT549 Cells to Carboplatin

**Figure 1:**
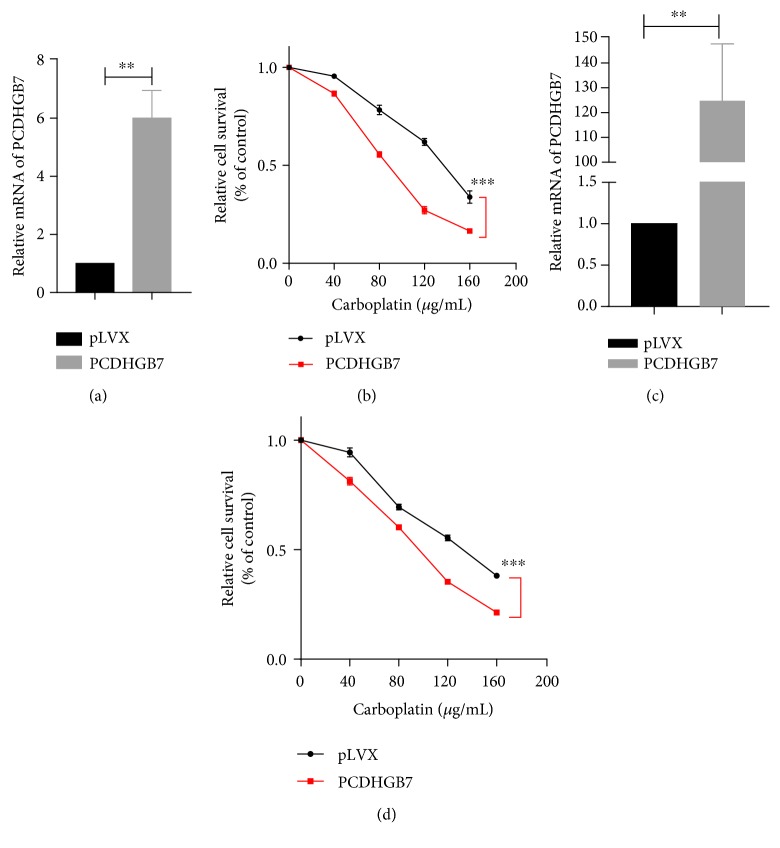
Overexpression of PCDHGB7 in breast cancer cells. (a, c) PCDHGB7 was overexpressed in Hs578T and BT549 cells (^∗∗^ *P* < 0.01). (b, d) Chemosensitivity assay in breast cells after treatment with different concentrations of carboplatin. PCDHGB7 overexpression and knockdown breast cancer cells were assessed by CCK-8 assay. Compared with Hs578T and BT549 cells, Hs578T and BT549 cells overexpressing of PCDHGB7 were more sensitive to carboplatin, respectively (^∗∗∗^ *P* < 0.001). Data are presented as the mean ± SD from three independent experiments.

**Figure 2:**
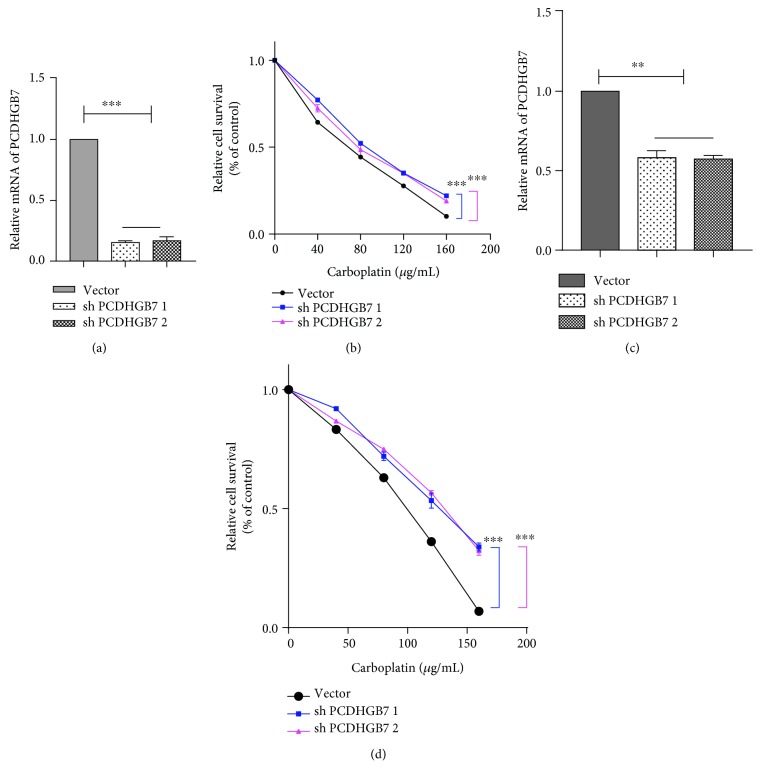
Depletion of PCDHGB7 in breast cancer cells. (a, c) PCDHGB7 was knocked down in Hs578T cells and BT549 (^∗∗^ *P* < 0.01). (b, d) Chemosensitivity assays in breast cells after treatment with different concentrations of carboplatin. Breast cancer cells with PCDHGB7 knockdown were assessed by CCK-8 assay. Compared with Hs578T and BT549 cells, Hs578T and BT549 cells with PCDHGB7 depletion were less sensitive to carboplatin, respectively (^∗∗∗^ *P* < 0.001). Data are presented as the mean ± SD from three independent experiments.

To investigate the roles of PCDHGB7 in breast cancer cells in vitro, pLVX lentiviral vectors were employed to overexpress PCDHGB7. PCDHGB7 mRNA levels in two breast cancer cell lines were verified in overexpressed and control cells (Figures 1(a) and 1(c)). Chemosensitivity was further confirmed by CCK-8 assay upon treatment of cells overexpressing PCDHGB7 with 40, 80, 120, and 160 *μ*g/mL of carboplatin. PCDHGB7 overexpression significantly increased apoptosis induced by carboplatin in Hs578T and BT549 cell lines (Figures 1(b) and 1(d), ^∗∗∗^ *P* < 0.001). eLCv2 lentiviral vectors were employed to knockdown PCDHGB7 in Hs578T and BT549 cells (Figures 2(a) and 2(c)). Apoptosis induced by carboplatin was remarkably reduced in Hs578T and BT549 cell lines (Figures 2(b) and 2(d), ^∗∗∗^ *P* < 0.001).

### 3.2. PCDHGB7 Inhibits HSPA9 Expression in Hs578T and BT549 Cells

**Figure 3:**
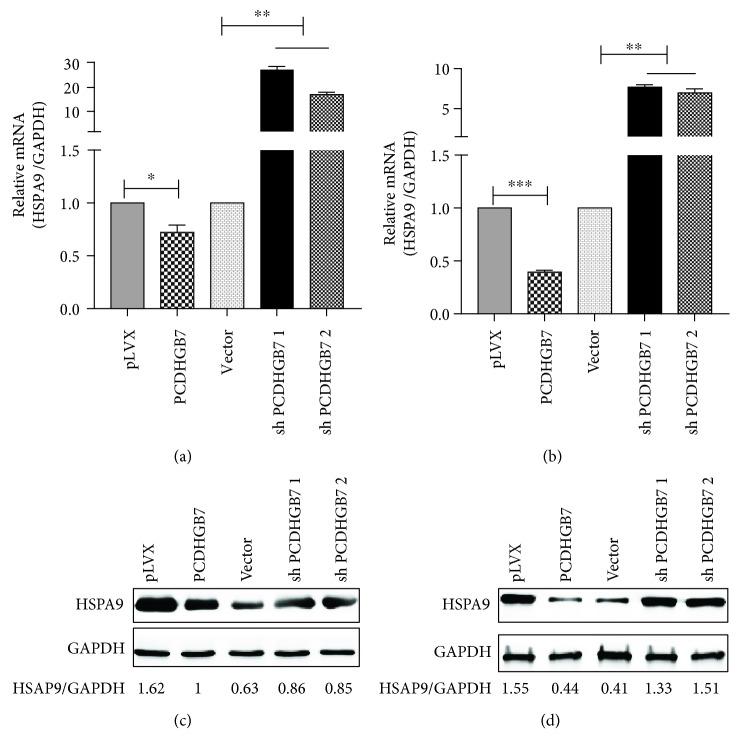
HSPA9 levels in Hs578T and BT549 cells with PCDHGB7 overexpression and PCDHGB7 knockdown. (a) HSPA9 mRNA levels are elevated in Hs578T cells with PCDHGB7 knockdown and reduced when PCDHGB7 is overexpressed. (b) The mRNA level of HSPA9 increased in PCDHGB7 knockdown BT549 cells and decreased in Hs578T cells with PCDHGB7 overexpression. (c) HSPA9 protein levels are elevated in Hs578T cells with PCDHGB7 knockdown and reduced in Hs578T cells with PCDHGB7 overexpression. (d). HSPA9 protein levels are increased in BT549 cells with PCDHGB7 knockdown and decreased in BT549 cells with PCDHGB7 overexpression. Data are presented as the mean ± SD from three independent experiments. The representative WB images are presented at the top panel (^∗^ *P* < 0.05, ^∗∗^ *P* < 0.01, and ^∗∗∗^ *P* < 0.001).

In our study, we also detected the HSPA9 mRNA levels in breast cancer cells (Hs578T and BT549) with PCDHGB7 knockdown and overexpression. HSPA9 mRNA levels were increased with PCDHGB7 knockdown and reduced with PCDHGB7 overexpression in both cell lines tested (Figures 3(a) and 3(b), ^∗∗^ *P* < 0.01 and ^∗∗∗^ *P* < 0.001). The protein levels of HSPA9 were affected in a similar fashion in both cell lines (Figures 3(c) and 3(d)).

### 3.3. PCDHGB7 Sensitises Hs578T and BT549 Cells to Carboplatin-Induced Apoptosis

**Figure 4:**
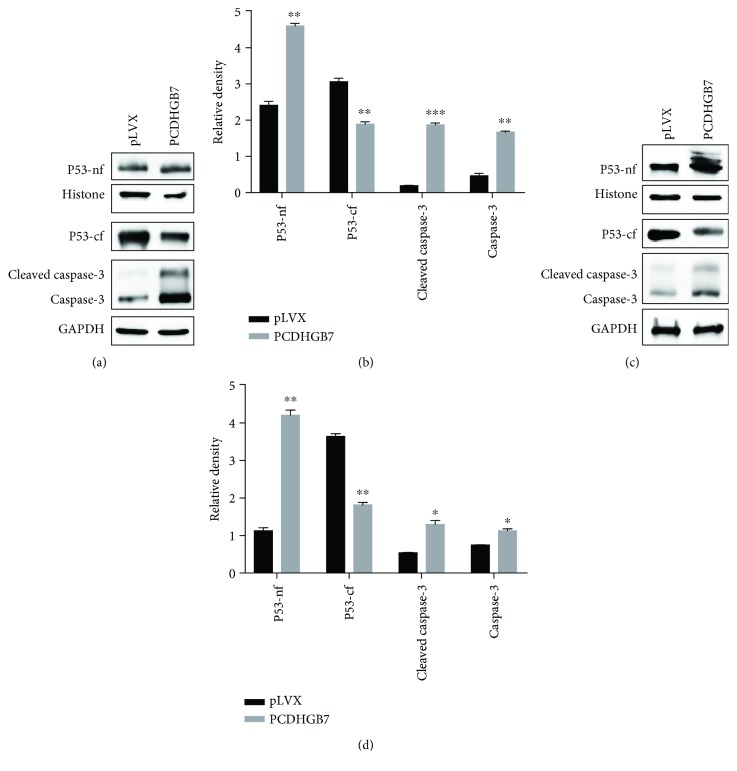
Western blotting analysis of expression of P53-nf (nuclear fraction of P53 and P53-nf), P53-cf (cytoplasmic fraction of P53 and P53-cf), cleaved caspase-3, and caspase-3 in PCDHGB7-overexpressing Hs578T and BT549 cells. PCDHGB7 protein is overexpressed in Hs578T cells (a, b) and BT549 cells (c, d). Cytoplasmic P53 (P53-cf) is significantly reduced, and nuclear P53 (P53-nf), cleaved caspase-3, and caspase-3 are elevated. Data are presented as the mean ± SD from three independent experiments (^∗^ *P* < 0.05, ^∗∗^ *P* < 0.01, and ^∗∗∗^ *P* < 0.001).

**Figure 5:**
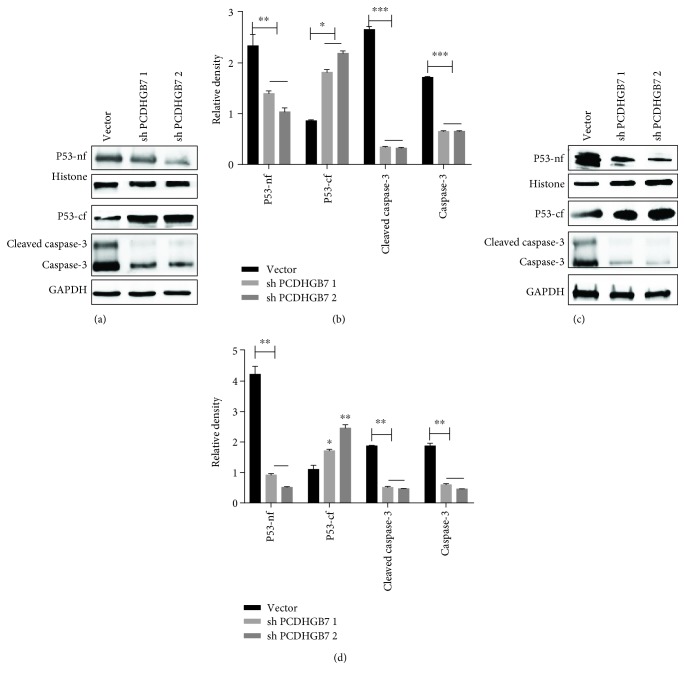
Western blotting analysis of P53-nf (nuclear fraction of P53 and P53-nf), P53-cf (cytoplasmic fraction of P53 and P53-cf), cleaved caspase-3, and caspase-3 expression in Hs578T and BT549 cells with PCDHGB7 knockdown. PCDHGB7 was knocked down in Hs578T cells (a, b) and BT549 cells (c, d). Nuclear P53 (P53-nf), cleaved caspase-3, and caspase-3 levels were significantly reduced, and cytoplasmic P53 (P53-cf) is elevated. Data are presented as the mean ± SD from three independent experiments. Representative WB images are presented in the top panel (^∗^ *P* < 0.05, ^∗∗^ *P* < 0.01, and ^∗∗∗^ *P* < 0.001).

Nuclear and cytoplasmic P53 and cleaved and uncleaved caspase-3 were detected in *Hs578T* and *BT549* cells treated with carboplatin. Overexpression of PCDHGB7 promoted P53 translocation to the nucleus and caspase-3 activation to induce apoptosis (Figure 4). On the other hand, depletion of PCDHGB7 inhibited P53 translocation to the nucleus and the inactivation of caspase-3 to suppress apoptosis (Figure 5).

### 3.4. HSPA9 Expression Inhibits Chemosensitivity to Carboplatin by Affecting Nuclear P53 Translocation and Apoptosis in Breast Cancer Cells

**Figure 6:**
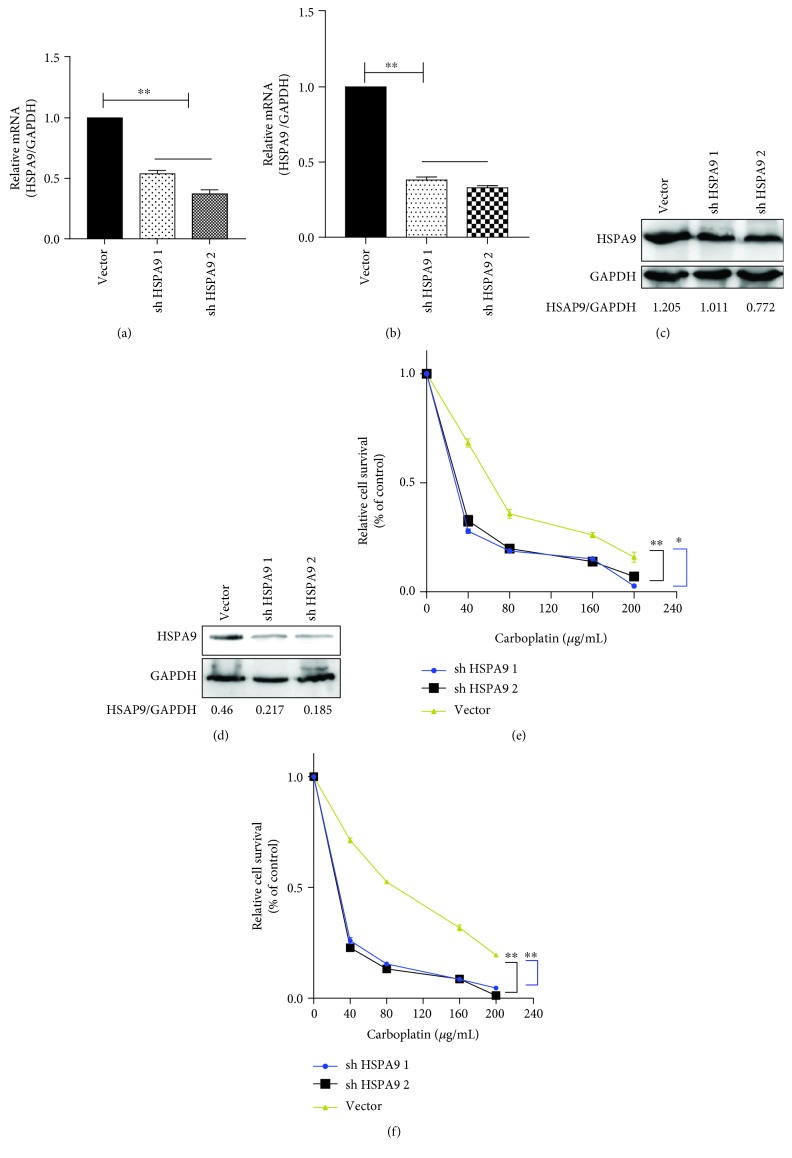
Construction of HSPA9 knockdown cell models in two BC cell lines, Hs578T and BT549, and the chemosensitivity assay in breast cells after exposure to different concentrations of carboplatin. The effects of HSPA9 knockdown were verified by real-time PCR and WB in Hs578T (a, c) and BT549 cells (b, d). (e, f) Carboplatin sensitivity in BC and HSPA9 knockdown breast cancer cells is assessed by CCK-8 assay. Chemosensitivity is significantly increased in Hs578T (e) and BT549 (f) breast cancer cells with abrogated HSPA9 expression. Data are presented as the mean ± SD from three independent experiments (^∗^ *P* < 0.05, ^∗∗^ *P* < 0.01).

**Figure 7:**
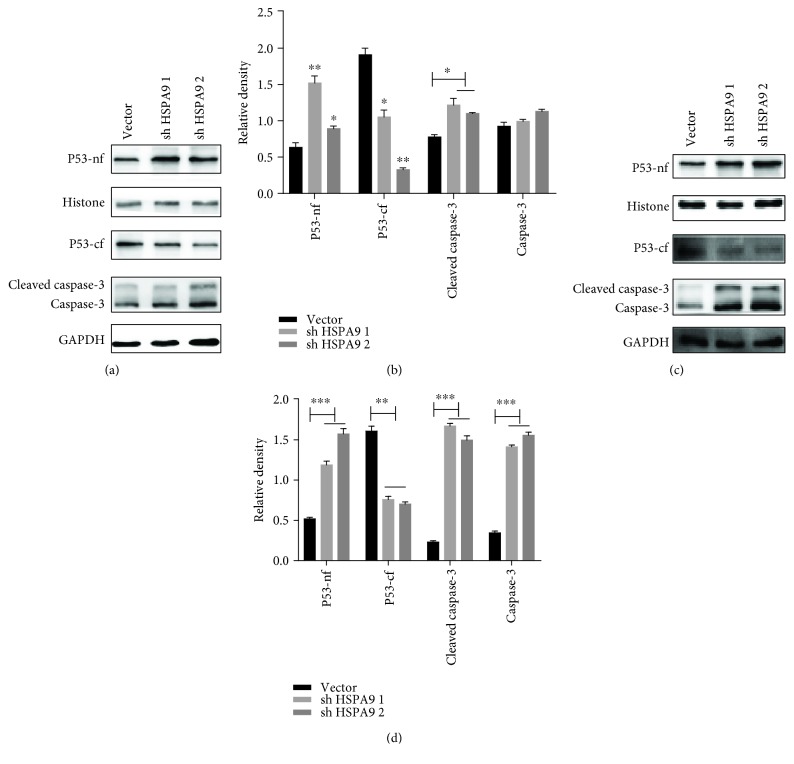
Western blotting analysis of P53-nf (nuclear fraction of P53), P53-cf (cytoplasmic fraction of P53), cleaved caspase-3, and caspase-3 in Hs578T and BT549 cells with HSPA9 knockdown. (a, b) P53-cf is significantly reduced in HSPA9-knocked down Hs578T cells, whereas P53-nf and cleaved caspase-3 are elevated. (c, d) P53-cf is decreased, whereas P53-nf, cleaved caspase-3, and caspase-3 are elevated in BT549 cells. Data are presented as the mean ± SD from three independent experiments. Representative WB images are presented in the top panel (^∗^ *P* < 0.05, ^∗∗^ *P* < 0.01, and ^∗∗∗^ *P* < 0.001).

To further investigate chemosensitivity after alteration of HSPA9 expression, we constructed Hs578T and BT549 breast cancer cells with HSPA9 knockdown (Figures 6(a)–6(d)). Increased chemosensitivity was observed with HSPA9 depletion in both cell lines (Figures 6(e) and 6(f)). Meanwhile, P53 translocation to the nucleus and the activation of caspase-3 were significantly increased (Figures 7(b) and 7(d); ^∗^ *P* < 0.05, ^∗∗^ *P* < 0.01, and ^∗∗∗^ *P* < 0.001), suggesting the antiapoptotic property of HSPA9 in the breast cancer cells.

## 4. Discussion

*Triple-negative breast cancer* (TNBC), a highly aggressive type of breast cancer with a poor prognosis and a high recurrence rate due to resistance to chemotherapy, accounts for 15-20% of all breast cancers [21–23]. It is defined by the absence of receptors for estrogen (ER), progesterone (PR), and epidermal growth factor receptor 2 (HER2) [24]. However, classifying a disease immunohistochemically by the absence of biological markers often has its limits. TNBC encompasses six heterogeneous subtypes of breast cancers, i.e., mesenchymal (M), mesenchymal stem-like (MSL), basal-like (BL1 and BL2), immunomodulatory (IM), and luminal androgen receptor- (LAR-) enriched tumors, each of which manifests distinct gene expression profiles and unique histological and molecular characteristics and responds differently to treatments [25].

Clinically, cytotoxic chemotherapy remains the standard treatment currently for patients in both early and advanced stages. Chemoresistance is often developed, and TNBC is characterized to have a high relapse rate within the first 3-5 years after completion of adjuvant therapy. Despite the efforts devoted to overcoming this challenge, the molecular mechanisms of the chemoresistance remain unclear, and the resolution is an urgent task [26, 27].

Our group had previously detected PCDHGB7 hypermethylation with low levels of HSPA9 in chemoresistant TNBC cells (unpublished data). In this study, we attempted to further investigate the roles of PCDHGB7 in the chemosensivity of TNBC cells. When overexpressed, PCDHGB7 significantly enhanced the apoptosis induced by carboplatin, a cytotoxic drug used in TNBC treatment, in both cell lines tested (Figures 1(b) and 1(d), *P* < 0.001). Conversely, both cell lines attained additional chemoresistance to carboplatin when the PCDHGB7 gene was knocked down (Figures 2(b) and 2(d), *P* < 0.001), confirming that PCDHGB7 positively enhances the chemosensitivity of breast cancer cells to carboplatin.

As a member of the protocadherin family, the biology of PCDHGB7 has been widely documented in the central nervous system [9] and in diseases such as non-Hodgkin's lymphoma [11]. However, the role of PCDHGB7 in TNBC is poorly understood thus far. This study is the first of attempts identifying PCDHGB7 as a proapoptotic protein in TNBC. The results from this study also suggest that low levels of PCDHGB7 might be responsible for the chemoresistance in some cases of TNBC.

In the present study, we also evaluated the HSPA9 levels upon overexpression/depletion and successfully established a significant negative correlation between the two at both the mRNA level and the protein level. Regrettably, the PCDHGB7 antibody was commercially unavailable at the point of study and the protein was only indirectly detected by the FLAG level in this study (supplementary figure 1).

Also known as mortalin or GRP75, HSPA9 is a member of the HSP70 family. It is mainly located in the mitochondria, where it plays a role in the ATP-dependent mitochondrial protein importing [28]. HSPA9 was also detected in the endoplasmic reticulum, Golgi network, exosomes, and plasma membrane, indicating that HSPA9 also has a role in endocytosis and exocytosis [29, 30]. Chemotherapy, radiation, and stress induce DNA damage, which triggers apoptosis by activation of P53, enhances the transcription of Bax, and inhibits the transcription of antiapoptotic genes, such as Bcl-2 [31]. HSPA9 leads to the inhibition of P53 transcription and apoptotic function via cytoplasmic sequestration [32]. Furthermore, UBXN2A (a p97-associated protein that is involved in protein quality control) and P53 share a common binding site on the HSPA9 protein. UBXN2A decreases the interaction between P53 and HSPA9; thus, considerably more P53 can be transported into the nucleus, which triggers P53-dependent apoptosis [33]. The colocalization of HSPA9 and P53 was detected in the perinuclear region in many types of cancer cells, such as HeLa (cervical carcinoma, wt P53), U2OS (osteosarcoma, wt P53), A172 (glioblastoma, wt P53), NT-2 (teratocarcinoma, wt P53), SY-5Y and YKG-1 (neuroblastoma, wt P53), MCF7 (breast carcinoma), and human adenocarcinoma cell lines [32]. Sequestration of P53 to prevent nuclear translocation enhances its degradation by MDM2 [34]. Prohibition of P53 sequestration in the cytoplasm by HSPA9 inhibitors may provide a novel therapeutic method for the treatment of a number of aggressive and drug-resistant cancers [35].

HSPs are thought to be an important component of the multifactorial, complex response of cancer cells to carboplatin. The relationship between the levels of expression of HSPs and sensitivity of cancer cells to carboplatin were reviewed previously [36]. An elevated level of HSPA9, with reduced caspase-3 and aberrant release of cytochrome c, has been reported to be responsible for the chemoresistance to cisplatin in breast cancer cells [35]. In light of this report, HSPA9 was depleted in Hs578T and BT549 cells before the cells were treated with carboplatin. In both cell lines, a low level of HSPA9 was observed to associate with significantly enhanced apoptosis (Figures 6(e) and 6(f), *P* < 0.001). We then proceeded to examine HSPA9 expression, P53 translocation, and caspase-3 activation upon treatment of carboplatin, in TNBC cells with either PCDHGB7 overexpression or depletion. It was observed that when cells had an elevated level of PCDHGB7, HSPA9 was significantly reduced and that these cells became significantly more susceptible to apoptosis induced by carboplatin. Such enhanced apoptosis was accompanied by P53 translocation and caspase-3 activation (Figures 4(a)–4(d)). Conversely, when PCDHGB7 was depleted, chemoresistance was observed and was shown to be associated with higher levels of HSPA9, sequestration of cytoplasmic P53, and reduced levels of both total and cleaved caspase-3 (Figures 5(a)–5(d)).

PCDHGB7 hypermethylation was previously detected in chemoresistant TNBC samples (unpublished data). In this study, we observed that the HSPA9 mRNA level was negatively regulated by PCDHGB7 (Figures 3(a) and 3(b)), strongly suggesting the involvement of PCDHGB7 in HSPA9 gene transcription. Further studies will be conducted to elucidate PCDHGB7's role in gene transcription in TNBC and to understand if PCDHGB7 methylation is responsible for the chemoresistance often found in TNBC treatment.

In conclusion, our study for the first time confirms that PCDHGB7 increases chemosensitivity of TNBC cells by negatively regulating the transcription and translation of HSPA9, rendering the breast cancer cells more susceptible to apoptosis via P53 translocation and caspase-3 activation. The study is also the first to report the novel role of PCDHGB7 in gene transcription in TNBC cells. Our findings present PCDHGB7 and HSPA9 as potential therapeutic targets for further studies in attempts to overcome chemoresistance in triple-negative breast cancer.

## Data Availability

The data used to support the findings of this study are included within the article.

## Supplementary Materials

Supplementary Materials Supplementary figure 1: overexpression of PCDHGB7 in breast cancer cells. Flag was overexpressed in overexpressing PCDHGB7 cells (^∗∗∗^ *P* < 0.001). Data are presented as the mean ± SD from three independent experiments.
