# Supplementary figures and images for: PCDHGB7 Increases Chemosensitivity to Carboplatin by Inhibiting HSPA9 via Inducing Apoptosis in Breast Cancer
Source: Dis Markers. 2019 Jul 8;2019:6131548. doi: 10.1155/2019/6131548 (PMC6652090; doi:10.1155/2019/6131548)

## Slide 1
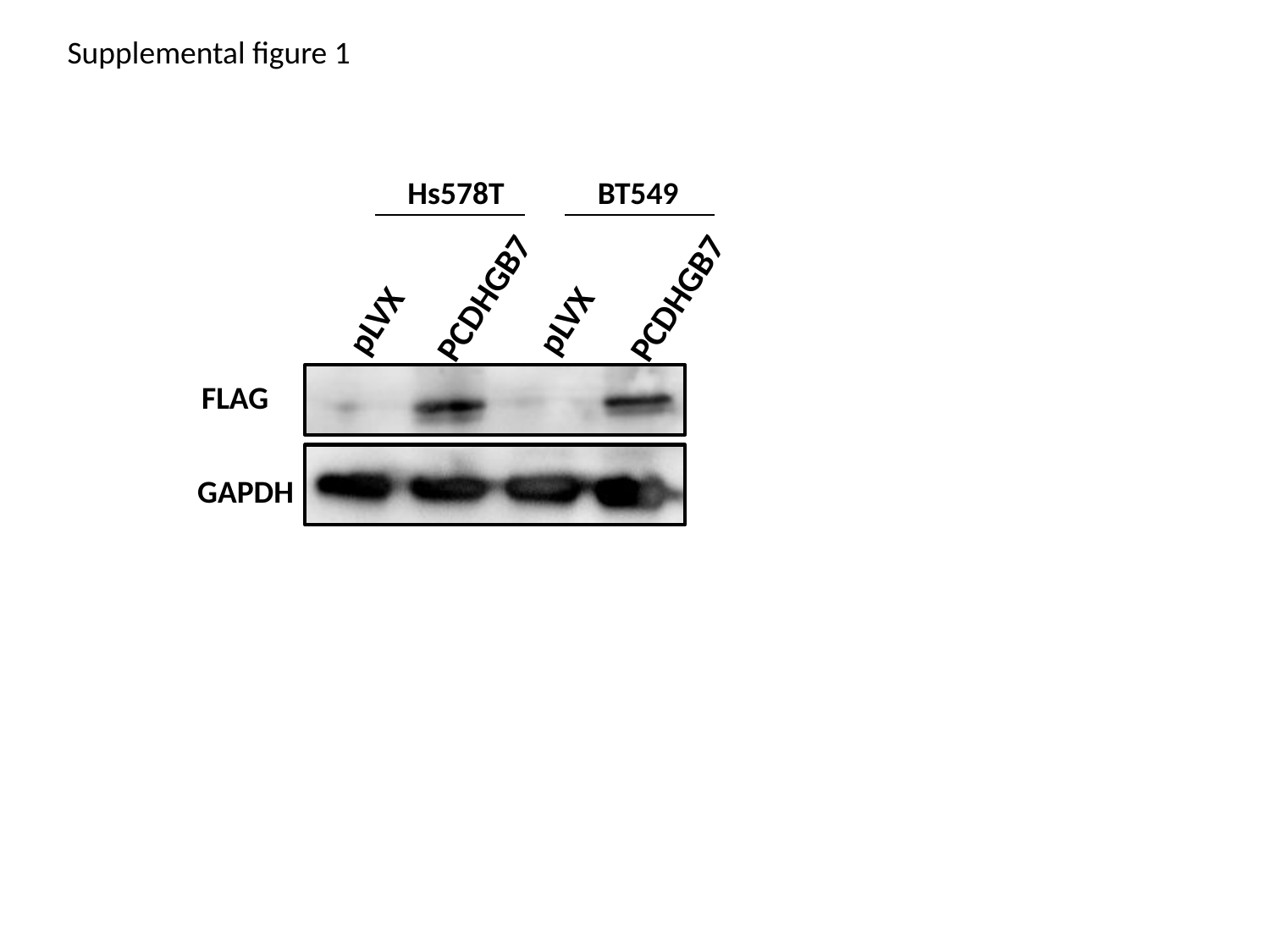

Supplemental figure 1
Hs578T
BT549
PCDHGB7
PCDHGB7
pLVX
pLVX
FLAG
GAPDH

Supplement: Supplementary Materials — Supplementary figure 1: overexpression of PCDHGB7 in breast cancer cells. Flag was overexpressed in overexpressing PCDHGB7 cells (∗∗∗ P < 0.001). Data are presented as the mean ± SD from three independent experiments. [file 6131548.f1.pptx]
